# Poverty-Related Diseases College: a virtual African-European network to build research capacity

**DOI:** 10.1136/bmjgh-2016-000032

**Published:** 2016-06-10

**Authors:** Thomas P C Dorlo, Carmen Fernández, Marita Troye-Blomberg, Peter J de Vries, Diana Boraschi, Wilfred F Mbacham

**Affiliations:** 1 Division Pharmacoepidemiology & Clinical Pharmacology, Utrecht Institute for Pharmaceutical Sciences, Utrecht University, Utrecht, The Netherlands; 2 Department of Pharmaceutical Biosciences, Uppsala University, Uppsala, Sweden; 3 Department of Molecular Biosciences, The Wenner-Gren Institute, Stockholm University, Stockholm, Sweden; 4 Department of Internal Medicine, Tergooi Hospital, Hilversum, The Netherlands; 5 Institute of Protein Biochemistry, National Research Council, Napoli, Italy; 6 Department of Biochemistry & Physiology, Faculty of Medicine, University of Yaoundé I, Cameroon

## Abstract

The Poverty-Related Diseases College was a virtual African-European college and network that connected young African and European biomedical scientists working on poverty-related diseases. The aim of the Poverty-Related Diseases College was to build sustainable scientific capacity and international networks in poverty-related biomedical research in the context of the development of Africa. The Poverty-Related Diseases College consisted of three elective and mandatory training modules followed by a reality check in Africa and a science exchange in either Europe or the USA. In this analysis paper, we present our experience and evaluation, discuss the strengths and encountered weaknesses of the programme, and provide recommendations to policymakers and funders.

**Key questions**

**What is already known about this topic?**

- Poverty-related diseases are a devastating health and socioeconomic problem in many low-income countries, particularly in sub-Saharan Africa.
- Traditional education and training in biomedical sciences usually do not offer perspectives on poverty and its related health conditions.

**What are the new findings?**

- We report our experience and evaluation of a unique international biomedical training programme that created a South-South and South-North network of young researchers, solely focused on poverty-related diseases.
- Poverty-Related Diseases College trained young biomedical scientists to become future trainers of the next generation of scientists in their own countries, addressing the needs of the poor in Africa.

**Recommendations for policy**

- Networks connecting researchers in disease-endemic countries and high-income countries are needed to build scientific capacity for poverty-related diseases and to conduct research across disciplines and diseases.
- Development of infrastructure, high-level education programmes and career promotion plans, supported by African home institutions and supervisors, are required for this.
- The sustainability of such networks largely depends on long-term funding.

## Training the future generation of African and European scientists in poverty-related diseases

Poverty is such a dominant factor affecting global health^1–3^ that the objectives of poverty alleviation programmes are usually strongly linked to health indicators.^4^ ^5^ This multidimensional concept of poverty is, for example, clearly expressed in the overlapping targets, goals and indicators of the recently proposed Sustainable Development Goals, defining the post-2015 health agenda.^6–8^ Poverty-related diseases (PRDs, see box 1) remain a devastating set of health and socioeconomic problems in many low-income countries, particularly in sub-Saharan Africa.^9^ Poor people do not only suffer from the greatest disease burden, but this burden is also given the least medical research attention.^10^ ^11^ Traditional education and training in biomedical sciences do not usually offer perspectives on poverty and its related health consequences.^12–14^ Training young researchers in the many aspects of PRD is therefore a pre-requisite for sustainable healthcare and development in developing countries. An additional major challenge is the poor career opportunities for researchers in Africa.^15–17^ As a consequence, young researchers often emigrate early in their scientific career, with little contribution to development of their native country. Strengthening health research capacity in Africa has been recognised as a key factor to address this, by funders and policymakers alike.^18–20^

Box 1 **Definition of poverty-related diseases (PRD)**

Many of the diseases contributing to the disease burden in low-income countries are tightly linked to the debilitating conditions of poverty, such as a lack of access to proper sanitation, health education and safe drinking-water, and poor nutrition and indoor air pollution. Diseases of poverty are often easily avoidable, preventable or treatable with existing medical interventions. According to the most recent Global Burden of Disease report by the WHO, 52% of the total disease burden in low-income countries is caused by PRDs.^21^ PRDs include the so called ‘neglected tropical diseases’, but also extend to a much wider spectrum of diseases and conditions causing high, though preventable, morbidity and mortality worldwide in low-income countries. They include, among others:

- HIV/AIDS
- Malaria
- Tuberculosis
- Parasitic diseases (eg, leishmaniasis, schistosomiasis, filariasis, trypanosomiasis)
- Other tropical diseases (eg, dengue, yellow fever, Buruli ulcer, leptospirosis)
- Treatable childhood diseases (eg, polio, measles, pertussis)
- Respiratory infections (eg, pneumonia)
- Diarrhoeal diseases
- Nutritional deficiencies
- Other perinatal and maternal conditions

The Poverty-Related Diseases College (PRDC) was a virtual African-European college created to give supplementary training to the doctors, health scientists and policymakers of tomorrow's developing world. PRDC aimed at enhancing exchange, and increasing knowledge and experience in PRD in order to build sustainable career opportunities for young biomedical scientists in Africa. PRDC merged theoretical teaching with hands-on experience in new skills and technologies, and provided this as a training programme to young researchers with different scientific backgrounds in Africa and Europe. The connection between basic and applied sciences was achieved by programming research electives within the context of the disease burden in Africa. The forging of South-North and South-South networks was considered an essential part of career development. The final and long-term goal was to build scientific capacity in poverty-related biomedical research in Africa, and to contribute to the development of highly motivated scientists embedded in and sustained by international networks. These scientists will act as the future trainers of the next generation of scientists in their own countries or contribute to health policies by their thorough understanding of fundamental and applied biomedical sciences in an African context.

The objective of this analysis paper is to present our experiences as an example of scientists and funders jointly inspired to develop sustainable scientific capacity and networks that address the medical needs of the poor in Africa.

## The Poverty-Related Diseases College

### Scope, structure and aims

**Figure 1:**
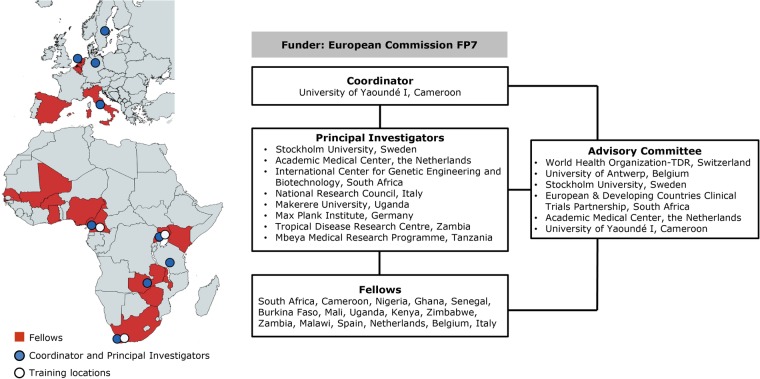
Structure of organisation of PRDC and origin of fellows and faculty. FP7, Seventh Framework Programme. PRDC, The Poverty-Related Diseases College.

The PRDC consortium was a virtual institute comprising five African and five European institutions providing a comprehensive training programme in scientific, technical and soft skills to a selected group of African and European biomedical research fellows. The structure of PRDC is shown in figure 1.

**Figure 2:**
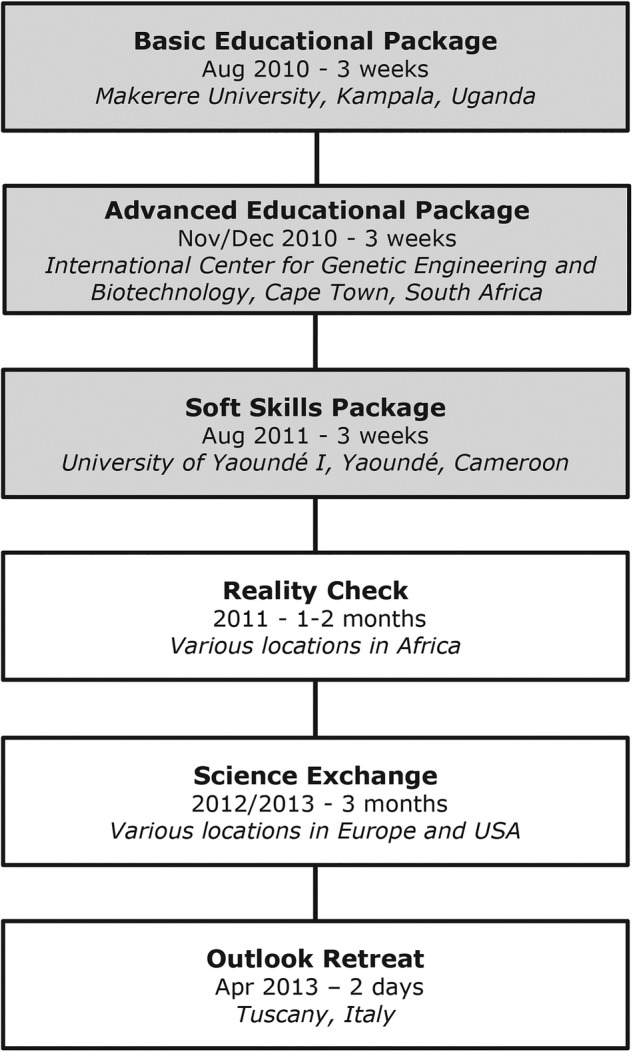
Overview of the various modules of the PRDC programme. PRDC, The Poverty-Related Diseases College.

The training programme consisted of a modular training curriculum (figure 2), preparing the fellows for a ‘reality check’ in Africa, followed by a science exchange programme at research laboratories in Europe and the USA and finalised by ‘outlook’ activities. The programme was guided by the following aims: (1) training and mentoring of future students by the fellows, (2) building sustainable research careers, (3) building networks around PRD, (4) specific training in advanced scientific technologies useful in PRD research and (5) improving cultural understanding.

### The funder

The programme was funded by the European Commission's FP7 within the funding scheme, Coordination and Support Action (call topic: ‘HEALTH-2007-2.3.2-14—Next generation of researchers for HIV/AIDS, malaria, tuberculosis and neglected infectious diseases’). The programme started in July 2009 and was completed in March 2013.

### The faculty

The faculty of PRDC was composed by an African coordinator, the University of Yaoundé I, Cameroon, and supported by principal investigators (PI) originating from five African research centres and four European institutions, all involved in different areas of scientific research and medical aspects of infectious-related and PRDs (see figure 1). The PIs undertook the task of organising and implementing the different joint courses, developing the topics and selecting the advisory group and the fellows. In addition, they facilitated the reality check, science exchange and outlook activities.

Six senior scientists with broad experience in research, teaching and administration, composed the advisory group from African and European institutions (figure 1). The advisors participated in the teaching and continuous contact with the fellows as well as with the PIs, supporting and advising on all modules of the programme.

### The fellows

In early 2010, following a competitive international open call for application, 24 fellowships were awarded to 16 African and 8 European young scientists, working in the area of PRD and affiliated to an academic institution as a student or as an employee. One additional African fellowship was awarded due to a drop-out during the programme. Selection criteria focused on personal talents and achievements (eg, publication record) but also included institutional backing of the potential fellow and their institutional supervisor's interests in the activities of PRDC, and required reference letters.

The fellows' countries of affiliation are shown in figure 1. Twenty-four per cent of African fellows were female, in contrast to 75% of the European fellows (total 40%). Among the African fellows, 11 were Anglophone versus 6 Francophone. The average age (range) was 28 (26–30) years and 35 (24–42) years for European and African fellows, respectively. Seventy-six per cent of the fellows had a MD or equivalent master's degree as highest degree at the time of enrolment, 5% a bachelor's degree and 19% (all of whom were African) had already obtained their PhD degree.

### Programme management

The programme was managed in accordance with all requirements of FP7 grant agreements. An unexpected and erroneous administrative interruption of the programme occurred during the organisation of the science exchange. This effectively resulted in a temporary arrest of forwarding funds from the funder and halted all activities for more than a year.

## Training activities in PRDC

### Courses

The PRD College offered three educational packages: (1) a ‘Basic Educational Package’, concerning theoretical biological, clinical, epidemiological and public health aspects of PRD; (2) an ‘Advanced Educational Package’, concerning practical technology mainly focused on modern immunological laboratory assays; and (3) a ‘Soft Skills Package’, focusing on research support, communication, administration, patents, grant applications and management, collaborations, gender balance, ethics, dissemination, public communication, philosophy and others (figure 2).

The three educational programmes were specifically designed to synchronise the educational level of the fellows so as to make them able to follow the longer and more demanding practical programmes.

### The reality check

An important pillar of the PRDC training programme was the ‘Reality Check’ module. In this module, hosted by the African institutions, the fellows were exposed to the reality of health and disease in low-income regions. The main aims were to train fellows in identifying determinants of health and health seeking in poor communities, and the responses of the care providers, regular and traditional, to existing health needs. To optimise the module, the fellows were grouped in teams mixing African and European fellows, which were operational at seven locations for a period of 1–2 months: Langa township, Cape Town, South Africa; Kati district, Mali; Mbeya, Tanzania; Chipulukusu, Ndola, Zambia; Malawi; Mbengwi, Cameroon; and Kasangati, Uganda. Fellows carried out 21 assignments ranging from ‘handshaking’ with local authorities, to collecting available local health data, conducting interviews with officials at various levels of health services, and organising focus group discussions to find out perceptions of diseases and opinions about the available healthcare systems among the population.

### The science exchange

The ‘Science Exchange’ activity consisted of 3-month training periods of the African fellows at European laboratories, in order to generate research data useful for their PhD projects, or for learning new skills and techniques, and experiencing different working and cultural environments. Owing to unforeseen administrative problems, this module was interrupted for an extensive period, demanding rescheduling of the training activities. Three fellows managed to conduct their training before the interruption, another eight fellows were able to conduct their training after the interruption and six fellows did not accept this opportunity, because they had continued their career in other directions. The overall output of the Science Exchange, despite the long interruption, was considered highly valuable, and turned out to be an important experience for all fellows involved.

### Outlook activities

The PRDC activities were concluded with a 2-day Outlook Retreat in Italy, where fellows, PIs and advisors reported their evaluation of the whole programme. Fellows were additionally asked to prepare joint grant proposals. These proposals were reviewed by the faculty and, based on their quality, six were allocated a small sum of money to develop a full grant proposal. On follow-up, three proposals were not developed due to individual problems of the fellows, and two were merged, given their common goals and participants. Thus, two grant proposals were finalised and submitted for funding. Neither was successful. One of them has been revised and resubmitted recently.

## Expectations and evaluations of PRDC

### Fellows' expectations and evaluations

Following the first course module, a semistructured questionnaire was developed to collect the fellows' viewpoints regarding their future career tracks and expectations from their enrolment in PRDC. The response rate to this questionnaire was 100%, and a descriptive and qualitative content analysis was performed on clustered answers.

At the time of enrolment, almost 90% of the African fellows had the expectation that PRDC would increase their chances on the job market compared to 50% of the European fellows. For the Africans, the main reason for joining PRDC was the scientific and technological content of the programme, while for the Europeans it was the possibility to expand their research collaborative network. Likewise, 80% of all fellows expected PRDC to change their view on PRD, mainly by linking a multitude of disciplines and backgrounds, and by increasing basic knowledge on, for example, pathophysiology and epidemiology of PRD. Most of the African fellows wanted to continue their research career after PRDC in their own country (50%) or to get limited training abroad and return to their country afterwards (12.5%), while most European fellows desired to use PRDC as preparation to network in an African context to advance their career in PRD (75%).

The majority of fellows found it more difficult to conduct research on PRD than to do so on other diseases, mainly due to limited funds. Possibly related to this, 50% and 37.5% of the African and European fellows, respectively, did not earn enough salary with their current research employment to make ends meet. Nevertheless, financial incentives to choose a professional direction were more apparent among African fellows.

Two years after the end of PRDC, in August 2015, another semistructured questionnaire was developed, to assess the impact of PRDC on personal and career development of the fellows. The response rate to this questionnaire was 88%, and a descriptive and qualitative content analysis was performed on clustered answers (the following percentages are based on the total number of respondents).

**Figure 3:**
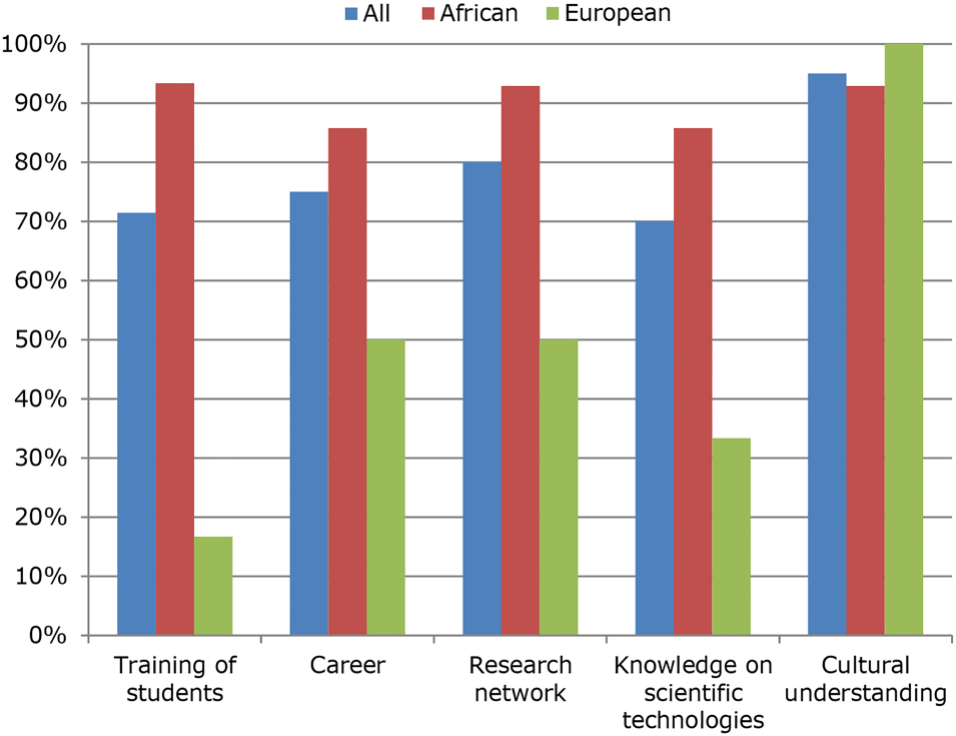
Impact of PRDC on various aspects of the fellows' career and personal development. The percentages indicate the proportion of positive impact evaluations. PRDC, The Poverty-Related Diseases College.

At the time of evaluation, 90% of the fellows had obtained their PhD degree. Most fellows remained in a research environment: 90% were employed by a university or university hospital, spending an average of 35% of their time in research and science, and 37% were in teaching. Most importantly, 81% of all respondents (93% of the African fellows) indicated that they were still involved in and actively pursuing research on PRD, in accordance with the PRDC's objectives. Their main research topics are summarised in table 1. In the 2 years after the end of PRDC, the fellows had published an average number of 4.5 journal papers per fellow (range 0–17). An average of 0.9 (range 0–6) papers per fellow was directly related to PRDC activities.^22^ The majority of fellows (67%) reported having transferred their PRDC training to their own students; for example, six fellows were supervising a total of 13 PhD students at the time of follow-up. Seven fellows had obtained grants during the follow-up period with a median value of €37 650 (range €10 000–346 623). When examining the outcomes versus the initial aims of PRDC (see section 2.1 above), ≥70% of the fellows found that PRDC indeed had a positive impact on all of those components (see figure 2). In general, the African fellows were more positive than the Europeans about the impact of PRDC on their careers. This might be explained by the fact that many aspects, such as teaching and knowledge on scientific technologies, were already appropriately covered in the curricula of the European graduate programmes. Nevertheless, almost all fellows (95%) indicated that PRDC had contributed to their mutual cultural understanding. Close interactions between all participants during all of the modules of PRDC and the frank scheduled discussions about encountered cultural differences contributed to the appreciation and acceptance of these differences. Many fellows explicitly acknowledged the positive impact of PRDC on their network and personal links (figure 3), but admitted that they had made little use of it in practice. While quantification remains difficult, the created networks did result in tangible results already within the (short) follow-up period: a South-South-North network established through the Science Exchange recently published the results of their collaboration in the *Malaria Journal*,^22^ and various PRDC fellows had been offered postdoctoral positions through connections made within PRDC.

**Table 1:** Current main poverty-related disease research interests of the PRDC fellows

Poverty-related disease	Number
HIV	4
Leishmaniasis	1
Hepatitis B/C virus	1
Lymphatic filariasis	1
Dengue	1
Malaria/helminth coinfection during pregnancy	1
Malaria	5
Vector-borne diseases	1
TB/HIV	1

PRDC, The Poverty-Related Diseases College; TB, tuberculosis.

The group dynamics among the different groups of fellows working and living closely together, showed rather normal and predictable features. Interpersonal dynamics sometimes adopted cultural issues, which were subsequently construed through focused group discussions. Although not explicitly measured, the focus on soft skills probably helped the fellows to use their enhanced communication skills to solve encountered problems.

### Advisors' evaluation

The advisors were asked to evaluate the project and to provide their opinion and personal experience of it. All declared that, in spite of certain administrative problems, the PRDC achieved its goals; and that the project was a very positive experience on a personal level. In addition to the main positive aspects, good spirit, motivation and dedication of both, fellows and faculty contributed to effective exchange of ideas and solutions to problems. The advisors encouraged the faculty to continue following and supporting the career of the fellows, and suggested creating an alumni association to provide means and incentives for maintaining contact between fellows. It is worth mentioning that the University of Yaoundé I has converted the PRDC curriculum into a full graduate programme.

## Conclusion and discussion

### General conclusion

The PRDC programme shows that it is possible to establish highly productive research and training networks that are based on international collaboration and inspiration. The programme was unique in its category, which warrants an analysis of its strengths and weaknesses. PRDC was also an impressive personal experience to fellows, faculty and advisors. It brought together, for almost 4 years, young and senior scientists coming from a number of different countries in Africa and Europe, with diverse scientific and cultural backgrounds, but who were invariably inspired to conduct research on PRD. The aims of PRDC in relation to the personal ambitions and expectations, assessed in the presented analysis, were largely fulfilled, with generally positive evaluations by the fellows (figure 3). The programme partially failed, however, to create a sustainable formal continuation of the created scientific network.

### Weaknesses, threats and suggested solutions

It proved difficult to implement a dynamic and efficient training programme within the context of ongoing and highly variable PhD tracks. In fact, the programme spanned 3 years (prolonged to four), during which time the students followed their individual PhD or postdoctoral paths at their home institutions. This sometimes gave rise to problems with their supervisors, because of the interruptions in their PhD projects while attending the PRDC courses and initiatives. This problem seems intrinsic to every ‘interfaculty’ initiative and may possibly be overcome by allocating more resources to the programme, and to its capacities to synchronise with existing PhD programmes. A more comprehensive research period in Africa, better integrated and focused on the PhD topic/programme of the fellow by involving the supervisors and departments at early planning stages of the fellows' activities, may have helped to make both programmes more compatible.

On the African side, a major issue was the limited infrastructure, especially with regard to laboratory equipment and reagents. It is imperative to persuade funders to develop grants that specifically include infrastructure development in African institutions. Another issue was the encountered gender imbalance, which could have been addressed by a quota for female fellows in the selection procedure.

The unforeseen and erroneous arrest in forwarding funds resulted in the need to suspend the entire programme for almost 1 year. This interruption resulted in the loss of involvement of some of the fellows, who had to continue their careers outside PRDC and were much less available once the programme resumed. Such unforeseen calamities can affect all programmes depending on external funding but the conditions of this error were typical of programmes that are carried out in and with developing countries. To prevent damage, commitment of the funder is needed to quickly repair administrative errors that threaten the continuation of the programme.

### Future perspectives

The future of PRDC has two main aspects: the individual future career development of its fellows, and the development of the PRDC concept into new initiatives. To maintain the network and increase its efficacy, the advisors suggested establishing an alumni programme. The sustainability of such a programme would be increased by the effort in developing the PRDC concept into long-term training initiatives that could take up the PRDC training modules and intercultural exchange plans. The University of Yaoundé I has started a self-sustaining Masters graduate programme that reproduces the PRDC curriculum since 2013. A similar programme in Public Health Biotechnology was started at the University of Ibadan, Nigeria, in 2015. The ‘Reality Check’ module has been adopted in the Masters programme in Health Economics at the Catholic University of Cameroon in Bamenda. Such programmes could be linked and connected through existing networks, such as The Global Health Network.^23^ The aspects of South-South and North-South collaboration and exchange are considered the major positive outputs of PRDC, and these need to be maintained in future dedicated programmes.

PRDC has shown that careful forging of bonds between fellows and PIs is probably key to success in intercultural and interdisciplinary training programmes. By emphasising respect for transcultural and other differences, group dynamics become very positive and productive. Group dynamics are rarely used as an instrument for scientific training. The experience of PRDC shows its potential.

### Recommendations to policymakers and funders

There is an urgent need for effective and sustainable actions to build scientific capacity to address poverty-related topics in Africa. To achieve this, it will be essential to put in place a network between researchers in disease-endemic countries and researchers in the developed parts of the world, who together will implement research efforts across disciplines and diseases. This will require promotion of high-level training of African researchers and their career development in African institutions.

The promotion activities should be three-tiered:

1. Development of infrastructure. Targeted equipment and infrastructure grants are needed to upgrade African institutions to become internationally more competitive. Funders should have a clear and transparent plan of research support that will allow maintenance of the infrastructures of African research institutions, universities and hospitals, at a competitive level. Such efforts could be coordinated by, for example, the African Academy of Sciences.
2. Development of high-level educational programmes. The PRDC experience highlights the feasibility of implementing successful educational programmes that, in addition to technical training, aim at cross-continental, cross-cultural and cross-disciplinary networking and collaboration. This kind of experience is of key importance for future scientists from both, low-income and high-income countries.
3. Development of a career promotion plan, especially for African researchers, supported by African home institutions and supervisors with the aim to attract back researchers from the large pool of highly skilled African scientists living outside Africa, as well as to reduce future brain-drain.

Importantly, the sustainability of such a network, as it was developed within PRDC, is largely dependent on the continuation of funding and interest of the funding agencies, subject to their critical evaluation. In our opinion, this long-term funding perspective would be pivotal to maintaining the momentum that was created by PRDC among the fellows, the involved institutions and the global health community, to consolidate medical science and training on PRD in the development of Africa.
